# Synergy of R-(–)carvone and cyclohexenone-based carbasugar precursors with antibiotics to enhance antibiotic potency and inhibit biofilm formation

**DOI:** 10.1038/s41598-022-22807-8

**Published:** 2022-10-26

**Authors:** Oliver Riester, Pia Burkhardtsmaier, Yuna Gurung, Stefan Laufer, Hans-Peter Deigner, Magnus S. Schmidt

**Affiliations:** 1 Institute of Precision Medicine, Furtwangen University, Jakob-Kienzle-Strasse 17, 78054 Villingen-Schwenningen, Germany; 2 Institute of Pharmaceutical Sciences, Department of Pharmacy and Biochemistry, Eberhard-Karls-University Tuebingen, Auf Der Morgenstelle 8, 72076 Tübingen, Germany; 3 Tuebingen Center for Academic Drug Discovery and Development (TüCAD2), 72076 Tübingen, Germany; 4 Faculty of Science, Eberhard-Karls-University Tuebingen, Auf Der Morgenstelle 8, 72076 Tübingen, Germany; 5 EXIM Department, Fraunhofer Institute IZI (Leipzig), Schillingallee 68, 18057 Rostock, Germany

**Keywords:** Medicinal chemistry, Chemical modification

## Abstract

The widespread use of antibiotics in recent decades has been a major factor in the emergence of antibiotic resistances. Antibiotic-resistant pathogens pose increasing challenges to healthcare systems in both developing and developed countries. To counteract this, the development of new antibiotics or adjuvants to combat existing resistance to antibiotics is crucial. Glycomimetics, for example carbasugars, offer high potential as adjuvants, as they can inhibit metabolic pathways or biofilm formation due to their similarity to natural substrates. Here, we demonstrate the synthesis of carbasugar precursors (CSPs) and their application as biofilm inhibitors for *E. coli* and MRSA, as well as their synergistic effect in combination with antibiotics to circumvent biofilm-induced antibiotic resistances. This results in a biofilm reduction of up to 70% for the CSP rac-7 and a reduction in bacterial viability of MRSA by approximately 45% when combined with the otherwise ineffective antibiotic mixture of penicillin and streptomycin.

## Introduction

The increase in antibiotic resistance (AR) and the associated health problems represent one of the major medical challenges for the next decades. In 2019, approximately 4.95 million (64 per 100,000) deaths worldwide could be associated with AR, of which 1.27 million (16.4 per 100,000) deaths were directly attributable to infections with antibiotic resistant pathogens^1^. Even in the case of non-fatal infections with antibiotic resistant pathogens, particularly in the case of chronic courses of disease, patients must cope with considerable loss in quality of life. To counteract this, several approaches must be pursued simultaneously. For example, it is necessary to minimize the development of new AR by a more targeted use of current antibiotics with the help of more accurate diagnostic tools, while at the same time developing new antibiotics or adjuvants that can successfully treat existing resistant pathogens^2,3^. There are several known mechanisms by which ARs act, including alteration or protection of the target, direct inactivation of antibiotics, or reduction in intracellular drug concentration^4^. Furthermore, biofilm formation is known as a widespread defense mechanism that causes resistance or increased tolerance of bacteria to antibiotics or host response^5,6^ and is significantly involved in implant infections, cystic fibrosis or chronic wounds^7,8^. One compound class with promising potential as antimicrobial and anti-biofilm agents that can be used to combat ARs, particularly ARs caused by biofilm formation, are carbasugars^9,10^.

Nowadays, carbasugars are discussed in medicinal and pharmaceutical chemistry as promising glycomimetics with potentially broad antibacterial characteristics and advanced properties, especially in terms of metabolic stability and bioorthogonality^11–14^. As early as the 1960s, MacCasland et al. described the synthesis of various carbasugars, such as pseudo-talopyranose or pseudo-galactopyranose, which they referred to as pseudosugars, which term today also includes iminosugars and thiosugars^15,16^. Many more approaches followed, which have been summarized in various review articles to date, focusing on either more applied or synthetic applications and research^11,13,17,18^. Many of the reported synthetic approaches have in common, that they start from simple monosaccharidic structures such as glucosamine and use several sequential derivatization techniques resulting in a multistep synthesis with low to moderate overall yields^19–22^. In addition, these pathways often contain transition metal-catalyzed synthetic steps involving, for example, mercury salts, which can be problematic in scale-up and ultimately in the pharmaceutical application itself^9^. Therefore a more straightforward approach based on other starting materials such as cyclic monoterpenoids, which can be categorized as precursors for substituted cyclohexane derivatives is of great interest. Other strategies based on non-carbohydrate sources as starting materials such as cyclohexadiendiol^23^, norbornene^24^, iodobenzene^25^ or benzoquinone^26^ try to overcome these disadvantages and have already been researched.

Herein, we present the first synthesis of simple carbasugar precursors (CSPs) based on R-(–)carvone and cyclohexenone as starting materials. They are called CSPs as they are no exact mimics of natural monasaccharides, but are similar to them and can be further modified to match the definition of carbasugars. These CSPs were evaluated for their potential in biomedical applications. This includes their cytotoxicity to human cells, their effect on bacterial growth and biofilm formation, and their synergistic effect in combination with antibiotics against biofilm formation and antibiotic-resistant bacteria.

## Results

### Synthetic approach to carbasugar precursors

**Figure 1:**
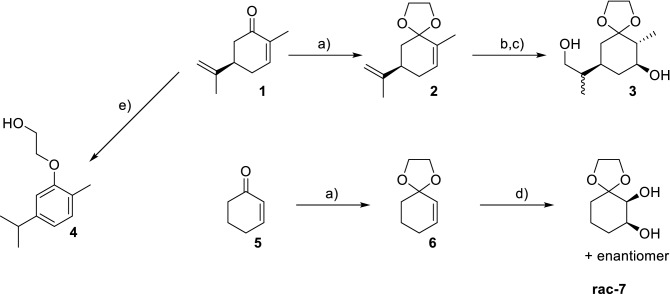
Synthetic approach to carbasugar precursors. (a) polyaniliniumsulfate, ethylene glycol, toluene, reflux (Dean–Stark apparatus); (**b**) BH_3_*THF, 2,3-Dimethyl-2-butene; (**c**) H_2_O_2_/NaOH; (**d**) KMnO_4_, MgSO_4_, H_2_O; (**e**) pTsOH or KHSO_4_ or NaHSO_4_, ethylene glycol, toluene reflux (Dean–Stark apparatus).

Many approaches towards carbasugar derivatives based on non-carbohydrate sources start, as mentioned in the introduction, from cyclohexene, cyclohexadiene or benzene derivatives, mainly because these allows the introduction of hydroxy groups by using various addition reactions, ideally stereo- and regeoselectively. During our research for an appropriate starting material we decided to use R-(–)carvone (**1**) because of the already present stereochemistry of the propenyl residue and the cyclic carbonyl group, which should allow the introduction of a substituted amino group later on via reductive amination. For related reasons we also chose cyclohexenone (**5**) as another starting material allowing the introduction of cyclic 1,2-diol functionalities. Based on the patent from Surburg et al*.*^27^ which describes the synthesis of compound **2** we initially tried to perform the ketal formation using KHSO_4_ or p-toluenesulfonic acid (p-TsOH). Figure 1 shows an overview of the used reaction conditions.

**Figure 2:**
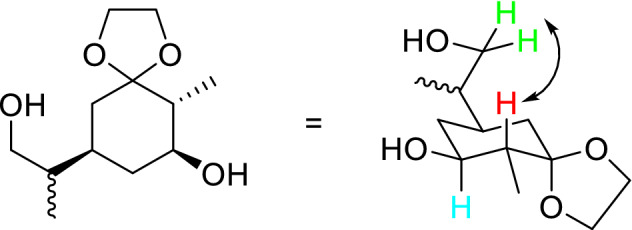
Chair conformation of compound **3** with indicated coupling in 2D NMR.

Additionally, to the use of the acids mentioned in the patent we also tried NaHSO_4_ as an alternative catalyst (conditions (e) in Fig. 1). Unfortunately, we were not able to reproduce the published results in lab scale (low gram quantities). Instead of compound **2** we were only able to isolate the literature known aromatic compound **4** as a sole main product in yields > 50% (spectral data agree with published data^28,29^). After additional literature research we decided to use polyanilinium sulfate as an alternative acidic catalyst based on the work of Palaniappan et al*.*^30^. The polyanilinium sulfate was synthesized according to the published data and after some optimization steps we succeeded in synthesizing compound **2** in isolated yields between 42–48%. For the stereoselective intramolecular bishydroboration we adapted the work of Brown and Pfaffenberger^31^ which used thexylborane for the cyclic hydroboration of dienes, especially of d-(+)-limonene, which has the same diene structure as compound **2**. After the optimization of the reaction, we could obtain compound **3** in yields around 40%. The trans configuration of the cyclic OH- and methyl-group could be confirmed by identifying the corresponding protons (see Fig. 2; red and blue colorized protons) via COSY (SI, Fig. S8) and by determination of the ^3^J_H,H_ coupling constant between these two protons, which, with a value of 10.5 Hz, correlates to the expected value estimated by the Karplus equation.

Furthermore, we were able to confirm the stereochemistry of the exocyclic alcoholic residue by NOESY (SI, Fig. S9) cross coupling between the green protons and the red in Fig. 2.

Unfortunately, we were not able to identify the stereochemistry of the exocyclic tertiary carbon. The fact, that the ^13^C spectrum (SI, Fig. S7) partially shows two very narrow signals for various carbons of compound **3**, indicates though, that we isolated a mixture of two diastereomers which presumably differ at the exocyclic tertiary carbon and cannot be identified or isolated by chromatographic techniques.

Comparing the method of Brown and Pfaffenberger with the use of simple borane BH_3_, it is interesting to mention that in case of using BH_3_, we only could obtain the exocyclic monohydroxy-compound selectively. The cyclic double bond has not been attacked by the borane.

For the synthesis of precursor **rac-7**, we started from compound **5** using polyanilinium sulfate as acidic catalyst resulting in compound **6** in 52% yield. Afterwards we used standard reaction conditions (conditions (d) in Fig. 1) for the diol formation leading to compound **rac-7** as racemic mixture in 30% yield. Compounds **3** and **rac-7** have then been used for the following biological evaluation.

### Biocompatibility

**Figure 3:**
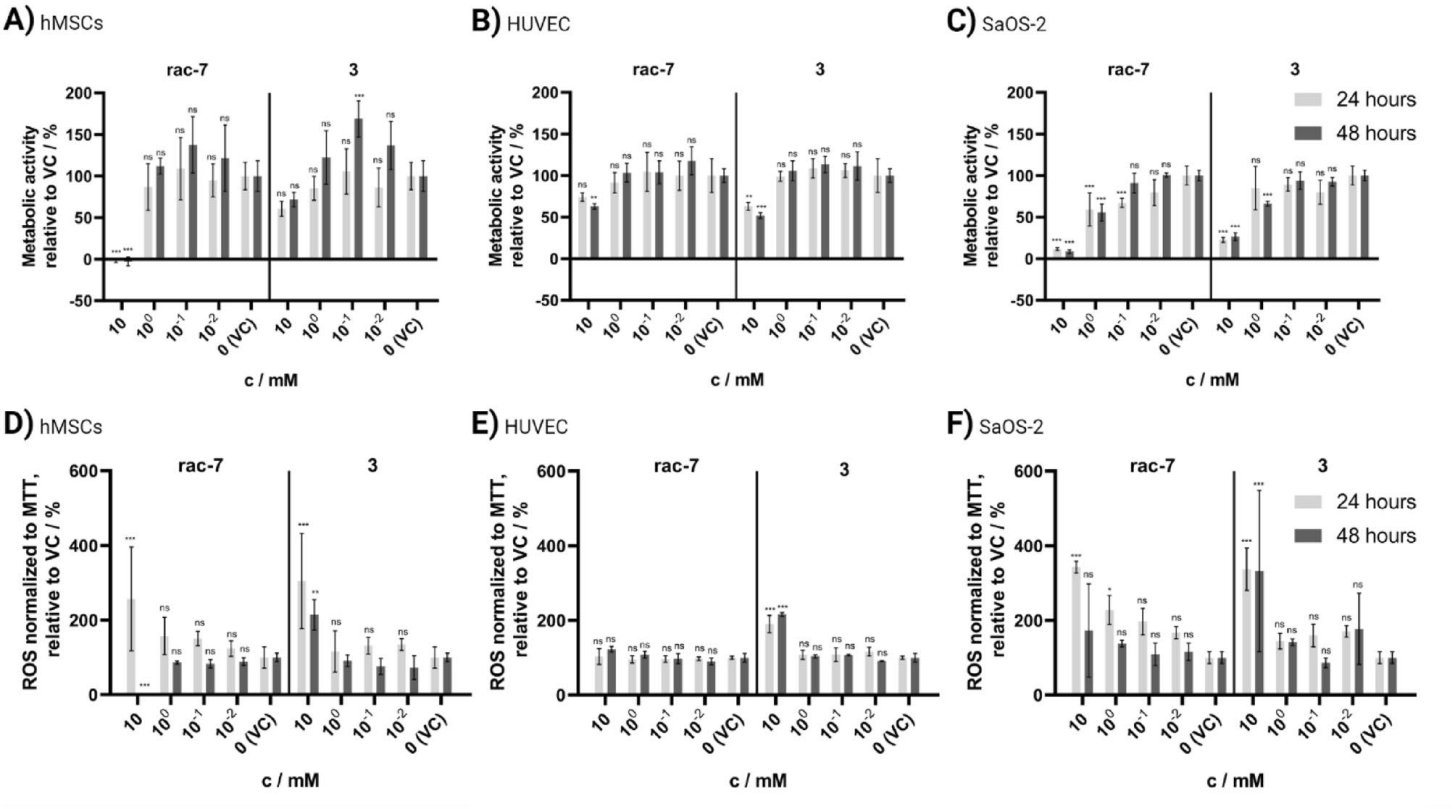
Biocompatibility of the compounds **rac-7** and **3**. Human mesenchymal stem cells (hMSC), human umbilical vein endothelial cells (HUVEC) and SaOS-2 human osteogenic sarcoma cells treated for 24 and 48 h. (**A**–**C**) Evaluation of metabolic activity at different concentrations of CSPs with MTT-assay in comparison to vehicle control (VC). (**D**–**F**) Cellular reactive oxygen species (ROS) normalized to MTT-assay and compared to VC. VC was treated with PBS instead of CSPs stock solution. Values are shown as mean ± SD (n = 3). Statistical significance was analyzed with Two-way ANOVA and Dunnett post-hoc test against respective VC (*ns* not significant; *p < 0.05; **p < 0.01; ***p < 0.001). For additional concentrations, as well as microscopic images, see Supplemental Fig. S1.

The potential for biomedical application depends fundamentally on the biocompatibility of the substance or material in question, otherwise the side effects of a treatment will exceed the benefits. To address this issue, we tested the synthesized CSPs for their biocompatibility with human cells. For this purpose, concentration-dependent metabolic activity and cellular reactive oxygen species (ROS) were determined for human osteogenic sarcoma SaOS-2 cells (SaOS-2), human bone marrow derived mesenchymal stem cells (hMSC), and human umbilical vein endothelial cells (HUVEC) (Fig. 3). No cytotoxic effects were observed for all three cell types at concentrations up to 100 µM (Fig. 3A–C), and a concentration of 1 mM proved toxic only for SaOS-2 cells, resulting in a reduction of metabolic activity for **rac-7** by up to 55 ± 9% (p < 0.001) and for **3** by up to 66 ± 2% (p < 0.001) compared to the control sample. However, the highest tested concentration of 10 mM resulted in strong cytotoxic effects in all cell types, resulting in metabolic activity of -3 ± 4% (p < 0.001), 63 ± 3% (p = 0.002), and 9 ± 2% (p < 0.001) for hMSC, HUVEC, and SaOs-2 cells for **rac-7** and 72 ± 7% (p = 0.46), 52 ± 3% (p < 0.001), and 26 ± 4% (p < 0.001) for **3,** respectively. This trend was confirmed by the measurements of cellular ROS level normalized to the metabolic activity (Fig. 3D–F), as seen by an increased cellular ROS level per metabolic activity with increasing concentrations of CSPs for hMSCs and SaOS-2 cells. HUVECs, on the other hand, seem to be less affected by the CSPs than the other two cell types, indicated by no significant increase of cellular ROS, except for 10 mM **3**. In addition, no significant change of cellular ROS level was observed for HUVECs between 24 and 48 h (Two-way ANOVA with Tukey post-hoc test, 24 against 48 h: p = 0.805 (**rac-7**); p = 0.9998 (**3**)), whereas a significant change over time was observed for hMSCs (Two-way ANOVA with Tukey post-hoc test, 24 against 48 h: p < 0.001 (**rac-7**); p < 0.001 (**3**)) and the compound **rac-7** for the SaOS-2 cells (Two-way ANOVA with Tukey post-hoc test, 24 against 48 h: p = 0.0021 (**rac-7**); p = 0.2312 (**3**)).

### Microbial growth

**Figure 4:**
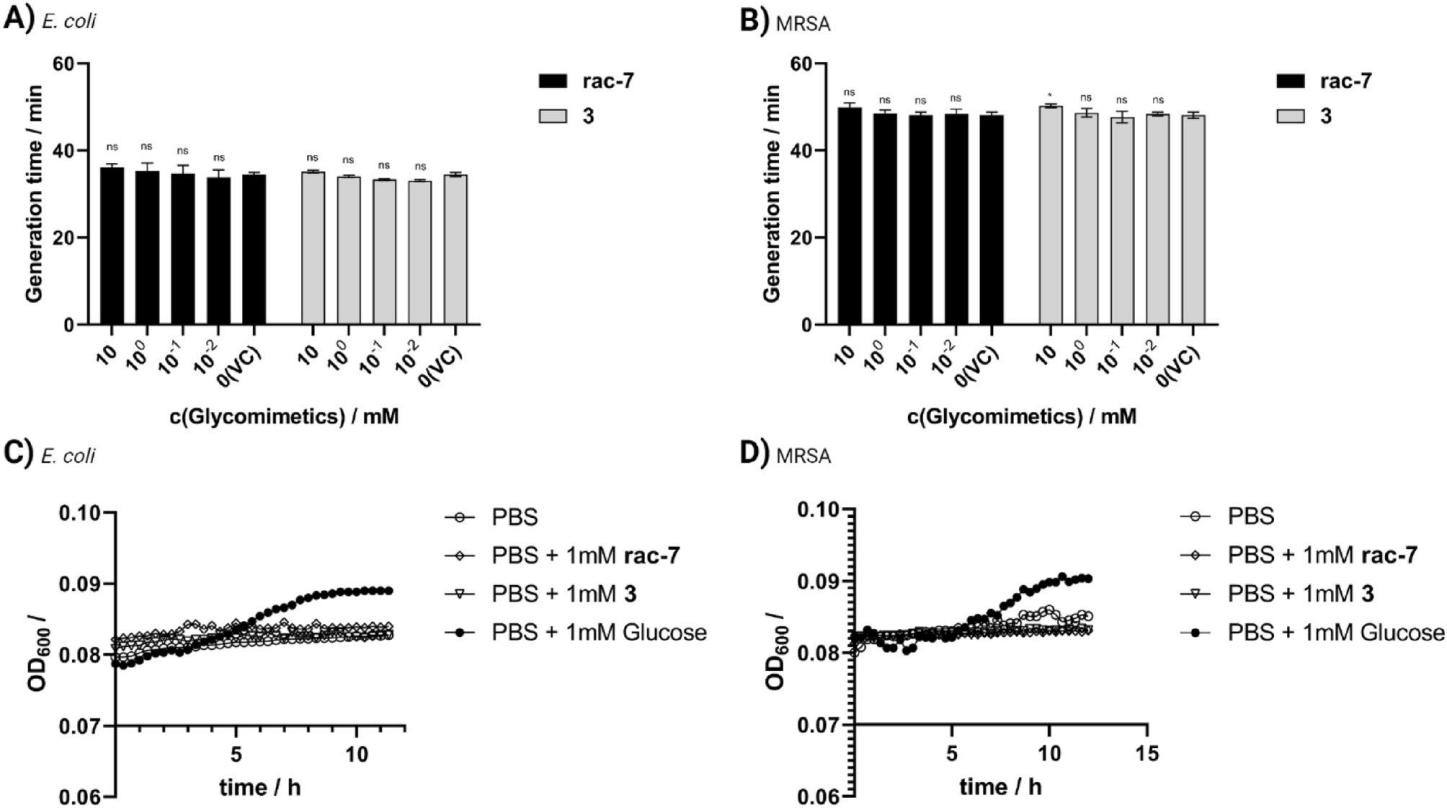
Effects of the compounds **rac-7** and **3** on the bacterial growth of *Escherichia coli* (*E. coli*) and multi-resistant *Staphylococcus aureus* (MRSA). Concentration-dependent influence of CSPs on the generation time of *E. coli* (**A**) and MRSA (**B**) in LB medium. Growth of *E. coli* (**C**) or MRSA (**D**) in PBS supplemented with CSPs or glucose. VC was treated with PBS instead of CSPs stock solution. Values are shown as mean ± SD (**A**,**B**: n = 3) or mean (**C**,**D**: n = 3). Statistical significance was analyzed with Two-way ANOVA and Dunnett post-hoc test against respective vehicle control (VC) (*ns* not significant; *p < 0.05; **p < 0.01; ***p < 0.001).

For biomedical applications, in addition to biocompatibility, the functional properties, particularly the antimicrobial properties, of the substance are important. Therefore, we investigated whether CSPs affect the growth of the gram-negative bacteria *Escherichia coli* (*E. coli*) and the gram-positive bacteria multi-resistant *Staphylococcus aureus* (MRSA) and whether they are used as a carbon source for growth. The growth of both bacterial strains in LB medium supplemented with the indicated concentrations of CSPs (Fig. 4A,B) differed only minimally or to a non-significant extent from the vehicle control without supplementation. Thus, no significant increase in generation time of *E. coli* was observed even for the highest tested concentration of 10 mM for both the compound **rac-7** with 36.1 ± 0.7 min (p = 0.35) and **3** with 35.1 ± 0.3 min (p = 0.95) compared to the vehicle control with 34.4 ± 0.4 min. For MRSA, a non-significant effect for **rac-7** with 49.9 ± 0.9 min (p = 0.15) and a significant effect for **3** with 50.3 ± 0.4 min (p = 0.05) on the generation time compared to the vehicle control with 48.1 ± 0.6 min was observed. Furthermore, it was observed that 10 mM **rac-7** leads to a considerable prolongation of the initial lag phase in MRSA growth (SI, Fig. S2A), which was not observed for *E. coli*, respectively compound **3** (SI, Fig. S2B–D). While there appears to be a concentration-dependent effect on bacterial growth, this effect is negligible and occurs only at concentrations that are not desirable for biocompatibility reasons (above 100 µM).

In addition, it was investigated whether the compounds **rac-7** and **3** are used as a carbon source for bacterial growth, which would indicate their metabolization. Our initial assumption that the CSPs could not be used as a carbon source was confirmed, as can be seen in Fig. 4C,D. No growth was observed in PBS or PBS with CSPs, but growth was observed when the same concentration of glucose was added.

### Biofilm

**Figure 5:**
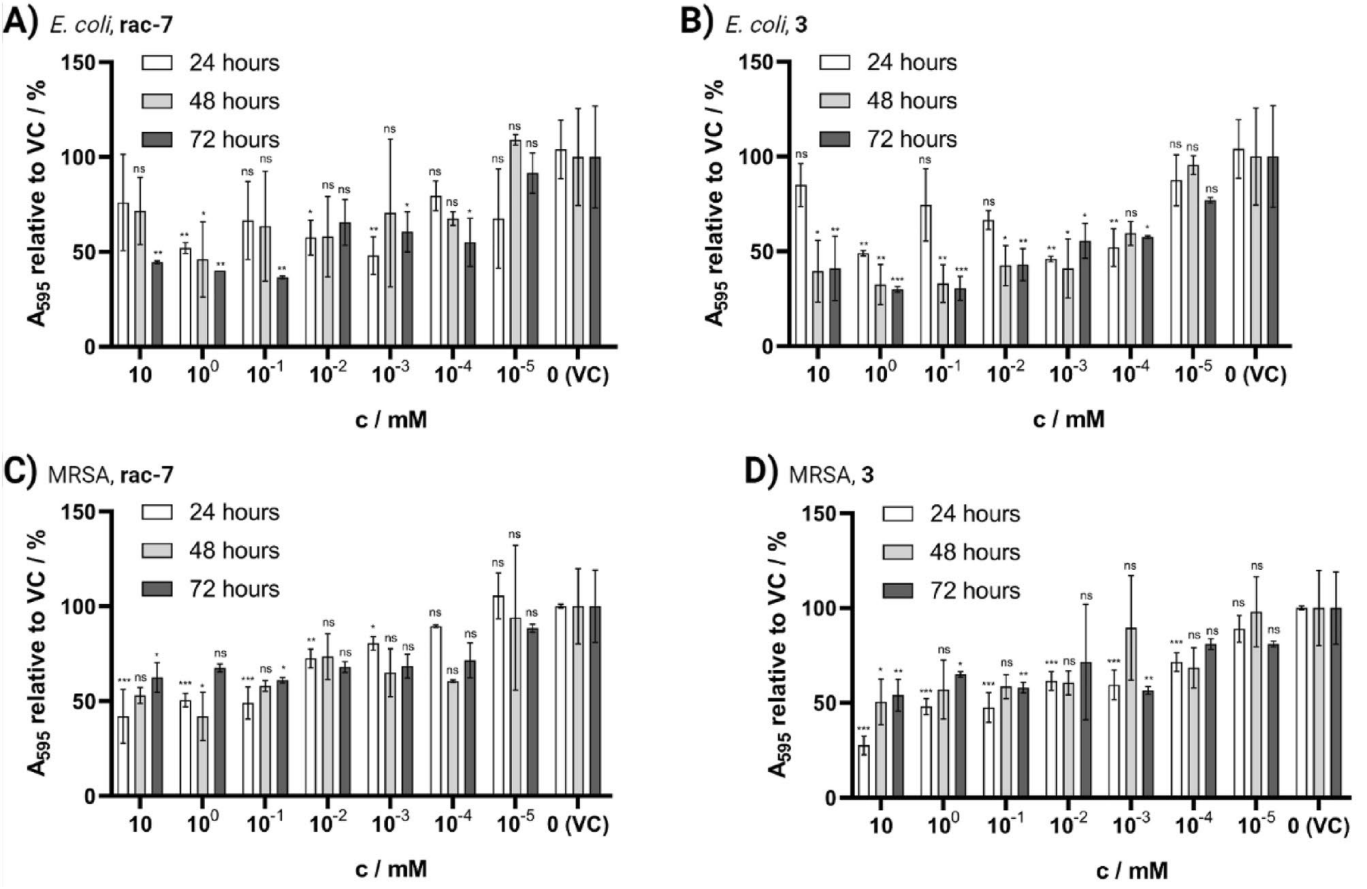
Evaluation of biofilm formation in the presence of CSPs with crystal violet (CV) assay. Biofilm formation of *Escherichia coli* (*E. coli*; **A**,**B**) and multi-resistant *Staphylococcus aureus* (MRSA; **C**,**D**) after 24, 48 and 72 h of incubation in the presence of 10 mM to 10 nM CSPs. Values are shown in relation to vehicle control (VC; PBS instead of compound stock solution) as mean ± SD (n = 2). Statistical significance was analyzed with Two-way ANOVA and Dunnett post-hoc test against respective VC (*ns* not significant; *p < 0.05; **p < 0.01; ***p < 0.001).

After investigating the effects on bacterial growth, we focused our attention on another important aspect of antimicrobial properties for biomedical applications: biofilm inhibition. Therefore, the biofilm formed in the presence of compounds **rac-7** and **3** was determined and compared with the vehicle control (VC) to which the same volume of PBS was added. In all combinations of the two compounds and bacterial strains, a significant decrease in biofilm after 24–72 h of incubation (Fig. 5) compared to the respective control was observed. There were only minor differences between the two compounds tested, most of which were within the range of the standard deviation. In addition, it is noteworthy that the compounds showed higher-relative-biofilm inhibition for the weak biofilm former *E. coli* (Fig. 5A,B) than for the good biofilm former MRSA (Fig. 5C,D), even though in absolute terms the biofilm formed by MRSA was more inhibited (SI, Fig. S3). Overall, 100 µM proved to be the concentration with the best compromise between good biocompatibility (Fig. 3) and biofilm inhibition for both compounds. Concentrations of 100 µM resulted in a reduction of biofilm formed by 64 ± 1% (**rac-7**, p = 0.001) and 70 ± 5% (**3**, p < 0.001) for *E. coli* as well as 39 ± 1% (**rac-7**, p = 0.02) and 42 ± 2% (**3**, p = 0.008) for MRSA after 72 h of treatment with 100 µM CSPs.

### Synergistic effect: carbasugar precursors and antibiotics

**Figure 6:**
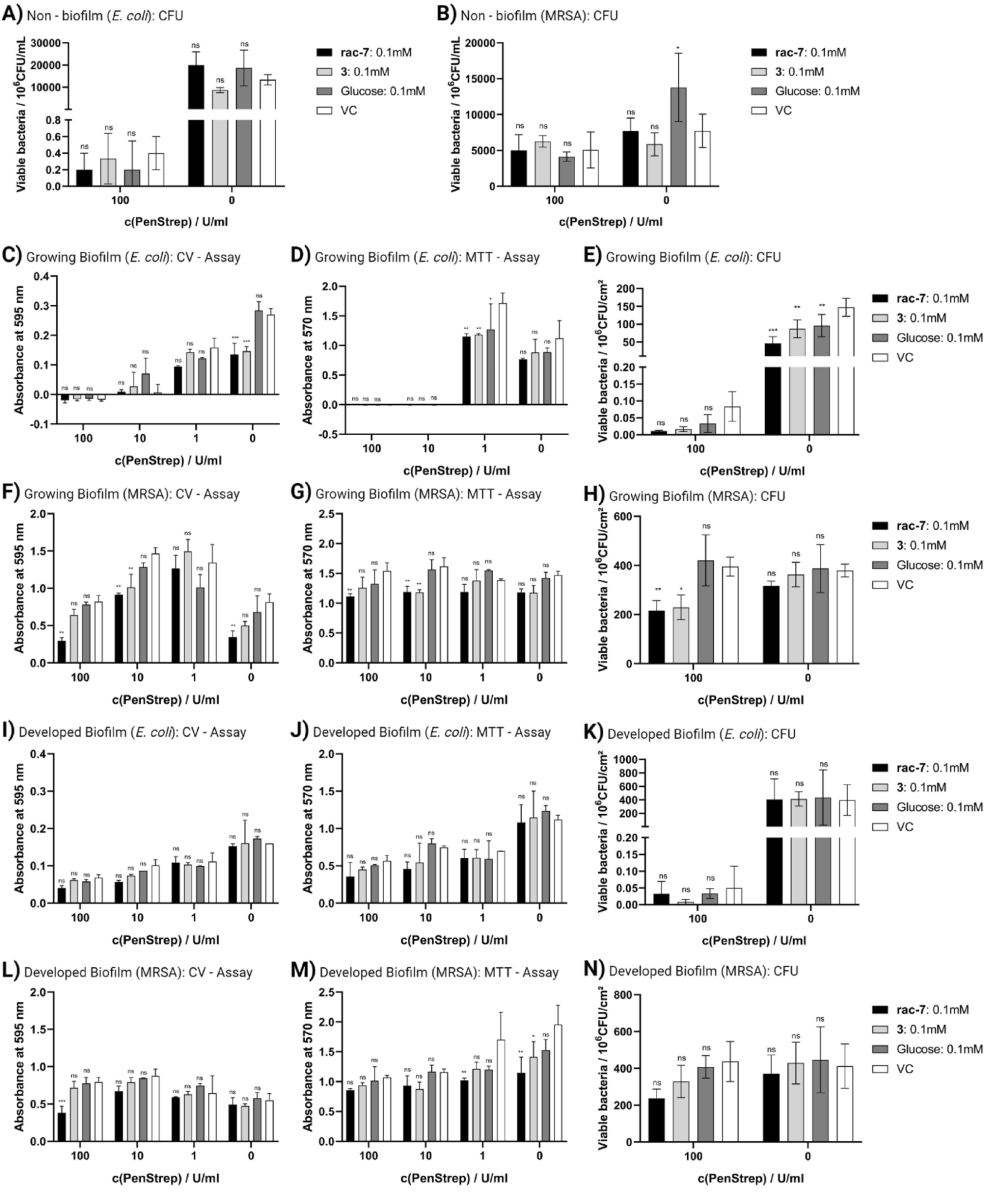
Synergistic effects of CSPs and antibiotics. Biofilm formation, metabolic activity and viable cells of *Escherichia coli* (*E. coli*; **A**,**C**–**E**,**I**–**K**) and multi-resistant *Staphylococcus aureus* (MRSA; **B**,**F**–**H**,**L**–**N**) under the influence of the compounds **rac-7** and **3** in combination with the antibiotics mixture of penicillin and streptomycin (PenStrep). Biofilm formation was evaluated via crystal violet (CV) assay (**C**,**F**,**I**,**L**), metabolic activity via MTT assay (**D**,**G**,**J**,**M**) and viable cells via CFU counting (**A**,**B**,**E**,**H**,**K**,**N**) after 48 h of treatment. Treatment was performed on non-biofilm cells (liquid culture in LB medium), growing biofilm and on developed biofilm. VC was treated with PBS instead of CSPs stock solution. Values are shown as mean ± SD (CV, MTT: n = 2; CFU: n = 3). Statistical significance was analyzed with Two-way ANOVA and Dunnett post-hoc test against respective VC (*ns* not significant; *p < 0.05; **p < 0.01; ***p < 0.001).

The formation of biofilms is a widespread defense mechanism of bacteria, not only as a protection against harsh environmental conditions, but also to reduce the effectiveness of antibiotics and increase bacterial tolerance. Therefore, it is important to prevent the formation of biofilm, e.g. in wounds or on surgical implants, to ensure promising treatment success for infections caused by multidrug-resistant microorganisms and to minimize the development of further resistance. We investigated a combinatorial approach of the synthesized CSPs in combination with the antibiotic mixture of penicillin and streptomycin (PenStrep) for synergistic effects. It was observed that *E. coli* was sensitive to PenStrep antibiotic mixture (Fig. 6A,E,K), while MRSA showed resistance (Fig. 6B,H,N) and even formed more biofilm at low antibiotic concentrations than without (Fig. 6F). Addition of PenStrep without CSPs to a growing biofilm or non-biofilm cultivation of *E. coli* prevented biofilm formation and resulted in nearly no viable cells remaining with no metabolic activity of the population. In contrast, the addition of PenStrep did not completely eliminate an already developed biofilm of *E. coli*, but significantly reduced it (Fig. 6I: Two-way ANOVA with Tukey post-hoc test; VC, 100 U/mL PenStrep against VC, 0 U/mL PenStrep: p < 0.001) and the number of viable cells (Fig. 6K: Two-way ANOVA with Bonferroni post-hoc test; VC, 100 U/mL PenStrep against VC, 0 U/mL PenStrep: p = 0.03), even though metabolic activity of the population could still be detected (Fig. 6J: Two-way ANOVA with Tukey post-hoc test; VC, 100 U/mL PenStrep against VC, 0 U/mL PenStrep: p = 0.01). The combined use of PenStrep with the compounds **rac-7** and **3** resulted for *E. coli* in a non-significant further reduction of the biofilm formed, metabolic activity and viable cells. As mentioned, the addition of PenStrep at low concentrations had a counterproductive effect on biofilm formation of MRSA, both in the developed biofilm and in the growing biofilm. The addition of CSPs was able to counteract this effect, such that when **rac-7** was added in combination with 100 µM PenStrep, biofilm formation was comparable to **rac-7** without PenStrep, but significantly less than 100 µM PenStrep without **rac-7** (Fig. 6F,L). However, the addition of CSPs, particularly **rac-7**, in combination with PenStrep (**rac-7** + PenStrep, 216 ± 33 × 10^6^ CFU/cm^2^) resulted in a significant reduction of viable bacterial cells (Fig. 6H) compared to separate addition (**rac-7**, 316 ± 16 × 10^6^ CFU/cm^2^; PenStrep, 395 ± 31 × 10^6^ CFU/cm^2^; VC, 379 ± 21 × 10^6^ CFU/cm^2^) when the compounds were present during the process of biofilm formation. A similar effect was observed for compound **3** on the viable bacterial cells (**3** + PenStrep, 229 ± 41 × 10^6^ CFU/cm^2^;** 3**, 362 ± 41 × 10^6^ CFU/cm^2^; PenStrep, 395 ± 31 × 10^6^ CFU/cm^2^; VC, 379 ± 21 × 10^6^ CFU/cm^2^). The addition of 100 µM **rac-7** in addition to 100 U/mL PenStrep reduced the biofilm formed by 64% (p = 0.003), the metabolic activity by 28% (p = 0.009) and the viable cells by 45% (p = 0.006) compared to the respective VC. Interestingly, **rac-7** also showed an effect on already developed biofilm of MRSA when administered in combination with 100 U/mL PenStrep (Fig. 6L), reducing biofilm by 52% (p < 0.001), cell viability by 46% (p = 0.1) but only non-significantly decreasing metabolic activity of the population with an absorption at 570 nm of 0.86 ± 0.02 (p = 0.59) compared to an absorption at 570 nm of 1.07 ± 0.02 for the corresponding VC.

## Discussion

The synthetic approaches for the mentioned CSPs, starting from simple and cheap compounds, turned out to be less trivial than expected. Especially the formation of the aromatic compound **4** seems to be problematic for the important first protection step of the keto-group of R-(–)carvone when using standard conditions like catalytic amount of pTsOH and a Dean–Stark apparatus. The use of polyanilinium sulfate helped to overcome these challenge, resulting in passable yields, which makes this catalyst very interesting for further studies using other simple natural products with predefined stereochemistry such as camphor or other terpenes containing keto-groups. Furthermore, intramolecular bishydroboration leading stereoselectively to compound **3**, has shown to be a very good tool on the way to stereoselective carbasugar synthesis.

For the biological evaluation, we demonstrate the synergistic effect of the compounds **3** and **rac-7** in combination with antibiotics against the bacteria *E. coli* and MRSA. When treated solely with the PenStrep antibiotic mixture, *E. coli* were susceptible and MRSA resistant to it (Fig. 6). When administered in combination against MRSA, the effects of PenStrep and the compound **rac-7** complemented each other and reduced both biofilm formation and viable bacteria beyond the level of single administration. For *E. coli*, no statement can be made about synergistic effects, since the addition of antibiotics alone had a strong effect, so that further effects could be barely detected. Thus, the proportion of biofilm present was reduced on the one hand, and the proportion of viable bacteria was reduced on the other hand, for both the already developed and the still growing biofilm, but not for the non-biofilm condition. Furthermore, it is worth mentioning that the reduction of viable cells was significant only for growing biofilm and not for developed biofilm, suggesting that the formation of new biofilm is inhibited, but existing biofilm is not actively disrupted. This demonstrates the potential of as adjuvants to antibiotics for applications where antibiotic resistance is expected as a result of biofilm formation, for example in open wounds, especially chronic wounds or after surgical implantation^32^. Various classes of substances with anti-biofilm properties have already been identified and tested for their suitability to inhibit biofilm, such as carbohydrate derivatives (e.g. derivatives of α-d-mannoside^10^, fucose or xylose^33^), d-amino acids^34^, polysaccharides (e.g. group II capsular polysaccharides^35^, chitosan derivatives^36^) or antimicrobial peptides^37^. These substances have different modes of actions and intervene at different stages of biofilm maturation, e.g., initial cell adhesion, secretion of extracellular polymeric substances, or biofilm degradation, as reviewed by Srinivasan et al.^38^ or Verderosa et al.^39^.

The mode of action of CSPs was not investigated in this study, but we hypothesize that they mitigate biofilm formation either through enzyme inhibition/protein binding, quorum sensing (QS) or a combination of both. Inhibition of biofilm formation by CSPs could occur through structural similarity to a natural substrate and act as a competitive inhibitor to cause enzymatic inhibition, as shown for example by Ren et al.^40^ for carbasugars and glycoside hydrolases, or cause reduced protein-target interaction, as shown by Sommer et al.^10^ for derivatives of methyl α-d-mannoside and the bacterial carbohydrate-binding protein LecB. A reduced protein-target interaction by competition for carbohydrate binding sites of lectins affects the bacterial adhesion of *E. coli* to surfaces, which in turn leads to inhibition of biofilm formation^41^. It is also possible that the CSPs activate QS systems: in gram positive MRSA, the accessory gene regulator (agr) system, as observed by Ueda et al. upon addition of 5.6 mM glucose and for gram negative *E. coli* the *N*-acyl l-homoserine lactone (AHL) mediated QS system^42,43^. Activation of the respective QS system leads to biofilm detachment and swarming motility, whereas repression of the agr system is necessary for biofilm formation^44,45^. Upon biofilm disruption, the bacteria leave the protective biofilm and enter the planktonic state, making them susceptible to antibiotics and human macrophages from which they were previously protected in the biofilm^6,46^. Moreover, unlike carbohydrates, the tested compounds have the advantage of not being used for bacterial growth (Fig. 4C,D), thus, on the one hand, they do not contribute to aggravation of the infection condition by bacterial growth and, on the other hand, they are present in a long-term effective concentration. Furthermore, the additional biofilm formed by MRSA as a defense response to PenStrep was significantly reduced when PenStrep was administered in combination with CSPs (Fig. 6F,L). Thus, the addition of CSPs not only allows circumvention of existing antibiotic resistance, but also ensures the efficacy of subsequent antibiotic treatments, as a more developed biofilm reduces the chances of success and even promotes the emergence of new antibiotic resistances^47^.

For the three human cell types tested, no toxicity was observed for concentrations up to 100 µM, nevertheless concentrations of 10 mM were toxic, especially for **rac-7**. Indeed, we observed that the CSPs had less toxic effects on HUVECs than on the hMSCs and SaOS-2 cells, which may readily be explained by an inhibition of glycolysis and differential dependence of cell types on it for ATP production. The energy metabolism of all three cell types is preferentially based on glycolysis and subsequent mitochondrial respiration, even in SaOS-2 cells, which does not show the Warburg effect typical for cancer cell lines^48–50^. The inhibition of glycolysis as a consequence of the high concentration of CSPs subsequently leads to a loss of ATP, both directly via the inhibition of glycolysis and indirectly by lowered generation of acetyl-CoA for the tricarboxylic acid cycle and NADH for oxidative phosphorylation. As an alternative energy source, cells are left with fatty acid oxidation (FAO) or glutaminolysis. In contrast to HUVECs, SaOS-2 cells and hMSCs have no significant FAO, which may be the reason that HUVECs showed higher tolerance to the tested CSPs (Fig. 3), since they can not only rely on glutaminolysis but also on FAO for ATP generation^51,52^.

The study presented here initially focused on the effect and applicability of the synthesized substances. The exact mechanisms leading to the toxic effects on human cells are interesting to rule out potential side effects, however, these only occur at very high concentrations of 10 mM, so they can be easily avoided in real conditions. In contrast, the exact mechanism for biofilm inhibition is of particular interest as it occurs at lower concentrations in the micromolar range, and by identifying the mode of action, novel glycomimetics can be functionalized in a targeted manner to achieve a better effect at even lower concentrations. Nevertheless, the presented study demonstrated the synergistic potential of CSPs or carbasugars in general to prevent biofilm-associated infections, supporting antibiotic treatments, and the possibility of reducing the impact of antibiotic resistance and the emergence of new resistances.

## Materials and methods

### Materials

Absorbance measurements were performed with a TECAN infinite M200 PRO (TECAN, Switzerland) plate reader. Microscopic images were taken with the microscope Olympus CKX41 (Olympus, Japan) mounted with the camera Olympus XM10 (Olympus, Japan) and the associated software cellSens Standard (Version 1.9 Build 11514, Olympus, Japan). 100 mM stock solutions of the compounds **rac-7** and **3** were prepared in phosphate buffered saline (PBS), sterile filtered with 0.2 µm nylon sterile filters (Carl Roth GmbH + Co. KG, Karlsruhe, Germany) and stored at 8 °C. T-75 cell culture flasks were purchased from VWR International GmbH (Bruchsal, Germany) and 96-well tissue culture plates were purchased from TPP Techno Plastic Products AG (Trasadingen, Switzerland). SaOS-2 human osteogenic sarcoma cells (SaOS-2, ACC 243) and human bone marrow derived mesenchymal stem cells (hMSC) were purchased from Sigma Aldrich (Taufkirchen, Germany) and human umbilical vein endothelial cells (HUVEC) were purchased from Fisher Scientific GmbH (Schwerte, Germany). 100 × penicillin–streptomycin mixture (PenStrep) containing 10,000 U/mL penicillin and 10,000 U/mL streptomycin was purchased from Lonza Group Ltd (Basel, Switzerland). Chemicals and reagents for the chemical synthesis were purchased from Acros, Sigma-Aldrich, Carl Roth or ABCR and were used without further purification. Chemicals and culture media for the biological evaluation were purchased from Sigma Aldrich (Taufkirchen, Germany) unless stated otherwise.

### Synthesis of carbasugar precursors

TLC was carried out on Silica Gel 60 F254 (Merck, layer thickness 0.2 mm) or on M&N RP-18 W/UV254 with detection by UV light (254 nm) and/or by charring with 15% sulfuric acid in ethanol. Flash column chromatography (FC) was performed on M&N Silica Gel 60 (0.063–0.200 mm) or on Macherey-Nagel (M&N) Silica Gel 100 C18 in case of RP-FC. ^1^H NMR and ^13^C NMR spectra were recorded either on a Bruker Avance 400 or on a Magritek Carbon 60. Chemical shifts are reported in ppm relative to solvent signals (CDCl3: δH = 7.26 ppm, δC = 77.0 ppm; DMSO-d6: δH = 2.49 ppm, δC = 39.7 ppm; CD_3_OD: δH = 4.78 ppm, δC = 49.3 ppm). Signals were assigned by first-order analysis and assignments were supported, where feasible, by two-dimensional ^1^H, ^1^H and ^1^H, ^13^C correlation spectroscopy. Coupling constants are reported in Hz. Electrospray ionization mass spectra (ESI) were performed on Sciex API QTRAP Mass Spectrometer (AB Sciex LLC, Framingham, MA, USA). The mass spectrometer was operated in the positive ion mode with an electrospray voltage of 5000 V at 200 °C, curtain gas at 25 psi, collision gas at 6 psi, nebulizing gas at 25 psi and auxiliary gas at 25 psi. All quadrupoles were working at unit resolution. RP-UPLC was performed on an Acquity system from Waters with a BEH C18 1.7 μm column (2.1 × 50 mm) from Waters and a gradient of water (eluent A) and methanol (eluent B) as an eluent. All compounds synthesized are > 95% pure by UPLC analysis.

#### Carvone-ketal (2)

10 g (66.6 mmol) R-(–)carvone, 11.1 g (179 mmol) ethylene glycol and 250 mg of polyanilinium sulfate were added to 100 mL of toluene in a 250 mL double-necked round bottom flask equipped with a Dean–Stark trap and a condenser. The mixture was heated to reflux for 8 h during the daytime and to 80 °C over night for a period of 4 days. Each day 2 more mL of ethylene glycol were added, and the reaction was monitored by TLC. After filtration over celite and purification by flash-chromatography (ethyl acetate/isohexane 9:1) compound **2** was isolated in 48% yield as a colourless oil. TLC: R_f_ = 0.35 (ethyl acetate/isohexane 9:1); RP-UPLC: *t*_r_ = 4.04 min (10–90% B in 7 min, 95.5% purity); ^1^H-NMR: (200.1 MHz, CDCl3) δ = 5.68 (m, 1 H, cycl. HC=C), 4.70 (m, 2 H, H_2_C=C), 4.10–3.87 (m, 4 H, OCH_2_CH_2_O), 2.43 (dddd, J = 13.6 Hz, 12.0 Hz, 4.8 Hz, 2.9 Hz, 1 H, CH_2_CHCH_2_),2.16 (dtt, J = 17.5 Hz, 5.3 Hz, 1,4 Hz, 1 H, 1 × CH_2_), 1.97–1.65 (m, 3 H, 3 × CH_2_), 1.71 (m, 3 H, CH_3_), 1.66 (m, 3 H, CH_3_); ^13^C-NMR (50.3 MHz, CDCl3) δ = 148.6 (C-8), 134.1 (C-5), 128.3 (C-4), 109.0 (C-9), 108.2 (C-6), 65.7 (C-12), 64.74 (C-13), 39.9 (C-2), 38.8 (C-1), 30.9 (C-3), 20.5 (C-10), 15.9 (C-7); (ESI-MS): m/z 195.3 [M + H]^+^.

#### Carvone-ketal diol (3)

10.4 mL (10.4 mmol) of a 1 M borane-THF complex solution and 1.3 mL (11 mmol) of 2,3-Dimethyl-2-butene were dissolved in 25 mL dry THF in a 250 mL Schlenk flask under nitrogen and stirred for 10 min. Then 2 g of compound **2** (8.7 mmol) was added and the mixture was stirred for additional 2 h at room temperature till no more starting material was detected by RP-TLC. Finally 1.1 g of NaOH dissolved in 1.8 mL of deionized water were added dropwise followed by the addition of 3.8 mL of 30% H_2_O_2_. After 2 h the mixture was diluted with 20 mL of deionized water and extracted several times with small amounts of ethyl acetate. The combined organic layers were washed with brine, dried (MgSO_4_) and the solvent was evaporated. After purification by RP-flash-chromatography (ethanol/water 1:9) compound **3** was isolated in 42% yield as a colourless oil. RP-TLC: R_f_ = 0.3 (ethanol/water = 1:9); RP-UPLC: *t*_r_ = 0.33 min (5–50% B in 7 min, 97.4% purity); ^1^H-NMR: (200.1 MHz, DMSO-d6) δ = 4.48 (d, J = 5.8 Hz, 1 H, cykl. OH), 4.34 (dt, J = 8.6 Hz, 5.1 Hz, CH_2_OH), 3.30–3.05 (m, 3 H, CH_2_OH, cycl. CHOH), 1.80–1.67 (m, 1 H, CHCH_2_OH), 1.65–1.31 (m, 3 H, 3 × CH_2_), 1.41 (dd, J = 10.5 Hz, 6.5 Hz, 1H, cycl. CH(CH_3_)), 1.15–0.91 (m, 2H, CH and 1 × CH_2_), 0.85 (d, J = 6.5 Hz, 3 H, cykl. CH_3_), 0,77 (dd, J = 6,5 Hz, 4.8 Hz, 3 H, CH_3_); ^13^C-NMR (50.3 MHz, CDCl3) δ = 110.7 (C-6), 72.1 (C-4), 65.3 (C-9), 64.6 (C-14), 64.5 (C-15), 47.8 (C-5), 37.3 (C-8), 33.0 (C-1), 32.6 (C-3), 14.1 (C-2), 13.5 (C-10), 10.1 (C-7); (ESI-MS): m/z 231.5 [M + H]^+^.

#### Cyclohexenone-ketal (6)

13.46 g (140 mmol) 2-cyclohexenone (**5**), 26.4 g (425 mmol) ethylene glycol and 130 mg of polyanilinium sulfate were added to 200 mL of toluene in a 500 mL double-necked round bottom flask equipped with a Dean–Stark trap and a condenser. The mixture was heated to reflux for 8 h during the daytime and to 80 °C over night for a period of 2 days. The reaction was monitored by TLC. After filtration over celite and purification by flash-chromatography (ethyl acetate/isohexane 1:5) compound **6** was isolated in 52% yield as a colourless oil. TLC: R_f_ = 0.46 (ethyl acetate/isohexane 1:5); RP-UPLC: *t*_r_ = 4.24 min (10–90% B in 7 min, 96.4% purity); ^1^H-NMR: (200.1 MHz, CDCl3) δ = 5.62 (m, 2 H, HC=CH), 3.98 (m, 4H, OCH_2_CH_2_O), 2.26 (m, 4 H, 2 × CH_2_), 1.75 (m, 2 H, 2 × CH_2_). ^13^C-NMR (50.3 MHz, CDCl3) δ = 126.5 (C-5), 124.3 (C-4), 107.9 (C-6), 64.3 (C-8 and C-9), 35.7 (C-1), 31.0 (C-3), 24.5 (C-2); (ES-MS): m/z 141.1 [M + H]^+^.

#### Cyclohexandiol-ketal (7)

5 g (35.7 mmol) of compound **6** were dissolved in 70 mL ethanol and cooled to 0 °C while a solution of 6.2 g KMnO_4_ and 3.1 g of MgSO4 in 70 mL deionized water was prepared and which was afterwards added dropwise to the reaction mixture during 30 min. After two more hours the mixture was filtered over celite and concentrated in vacuum to a volume of 80 mL. The concentrated aqueous mixture was saturated with NaCl and extracted a dozen times with CH_2_Cl_2_. Each extract was analyzed by TLC and extracts containing starting material or byproducts were dismissed. The combined product fractions were dried (MgSO_4_) and the solvent was evaporated. The residue was recrystallized from isohexane to yield compound 7 in 26% yield. TLC: R_f_ = 0.14 (ethyl acetate/isohexane 1:1); RP-UPLC: *t*_r_ = 0.33 min (5–50% B in 7 min, 95.4% purity); 1H-NMR: (200.1 MHz, CDCl3) δ = 4.08–3.86 (m, 5 H, CHOH and OCH_2_CH_2_O), 3.67 (ddd, J = 12.6 Hz, 5.3 Hz, 3.0 Hz, 1 H, CHOH), 2.51 (br. s, 2 H, 2 × OH) 1.99 (ddd, J = 13.7 Hz, 6.0 Hz, 2.2 Hz, 1 H, 1 × CH_2_CHOH) 1.86–1.43 (m, 5 H, CH_2_). (ESI-MS): m/z 197.2 [M + H]^+^.

### Cell culture

Biocompatibility assays were performed with three human cell types: SaOS-2 cells, hMSC and HUVEC. All three cell types were purchased from Sigma Aldrich (Taufkirchen, Germany). SaOS-2 cells were cultured in McCoy’s 5A medium supplemented with 10% fetal calf serum; hMSCs were cultured in stem cell expansion medium SCM015; HUVECs were cultured in endothelial cell growth medium. All cell culture media were supplemented with 100 U/mL PenStrep. Cells were cultured in T-75 cell culture flasks at 37 °C and 5% CO_2_ and subcultured at 80% confluency with 0.25% Trysin-EDTA solution. hMSCs and HUVECs were expanded for no more than 3 passages before use.

### Metabolic activity—MTT assay

Biocompatibility of CSPs was evaluated with SaOS-2 cells, hMSCs and HUVECs using 3-(4,5-dimethylthiazol-2-yl)-2,5-diphenyltetrazolium bromide (MTT) assay. Briefly, trypsinated cells were seeded into 96-well tissue culture plates at a density of 10,000 cells/well and incubated at 37 °C and 5% CO_2_ for 4 h in 180 µL of appropriate culture medium. Meanwhile, a serial dilution of the compounds **rac-7** and **3** stock solutions was prepared in sterile PBS and 20 µL of each was added to the respective well, resulting in effective concentrations of 10–10^–5^ mM. As a vehicle control, 20 µL sterile PBS without CSPs was used. Cells were cultured for another 24 or 48 h in the incubator. After 24 or 48 h, medium was replaced by 1 mg/mL MTT in culture medium and incubated for 2 h. Then, MTT solution was removed and wells were washed three times with 200 µL sterile PBS. By adding 100 µL DMSO per well and incubation at 37 °C for 1 h, the formed formazan crystals were dissolved and subsequently quantified at 570 nm in a plate reader. Metabolic activity was calculated as the ratio of absorbance between samples and the vehicle control.

### Cellular reactive oxygen species—DCFDA assay

Cellular reactive oxygen species (ROS) were analyzed by 2′,7′-dichlorofluorescin diacetate (DCFDA) assay for SaOS-2 cells, hMSCs and HUVECs. Cells were prepared as described for the MTT-assay, resulting in cells treated for 24 or 48 h with 10–10^–5^ mM CSPs. As additional controls, the ROS quenchers *N*-acetylcysteine (NAC) or butylated hydroxyanisole (BHA) were added to indicated controls to a final concentration of 250 µM or 5 µM, respectively. After 24 or 48 h of treatment, 2 µL 2 mM DCFDA in culture medium was added to each well and incubated at 37 °C for 30 min. Culture medium was removed and cells were washed three times with 200 µL sterile PBS. Oxidized dye was quantified with a plate reader at Ex/Em wavelengths of 485 nm/535 nm.

### Microbial evaluation

Gram negative *Escherichia coli* (DSM 498) and gram-positive multi-resistant *Staphylococcus aureus* (MRSA, DSM 28766) were purchased from Leibniz Institute DSMZ-German Collection of Microorganisms and Cell Cultures GmbH (Braunschweig, Germany) and stored at − 80 °C in glycerol stocks. A new vial was thawed for each experiment and incubated overnight at 37 °C and 100 rpm in Lennox LB medium.

### Bacterial growth

Bacterial growth in PBS or LB medium was recorded in 96-well plates. Therefore, 180 µL of sterile PBS or LB medium containing the appropriate concentration of CSPs or glucose was added to each well and inoculated with 50,000 bacteria/well to reach a total volume of 200 µL. The lid of the 96-well plate was closed and sealed with parafilm. OD_600_ was measured every 30 min in a plate reader for a total of 16 h at 37 °C without shaking. The logarithmic growth curves were used to calculate bacterial generation time. For this purpose, a linear regression of the exponential growth phase was performed with GraphPad Prism 8 (GraphPad Software, San Diego, USA) and the slope k was determined. Then, generation time (t) was calculated according to Eq. (1).

1 $$t=\frac{ln2}{k}$$

### Sample preparation and treatment

Non-biofilm bacteria growing in liquid broth were prepared by adding 2 mL of LB medium to a sterile 15 mL conical plastic tube, and the appropriate volume of PenStrep and compound stock solution or sterile PBS, as vehicle control, were added. Samples were inoculated with 20 µL of an bacterial overnight culture diluted to OD_600_ = 0.1, resulting in approximately 10^5^ CFU/mL, and incubated in a humidified incubator at 37 °C and shaking (130 rpm) for 48 h before analysis by spot plating as described below.

Developed biofilm was prepared in 96-well plates by incubating 200 µL of an bacterial overnight culture diluted to OD_600_ = 0.2 with LB medium at 37 °C in a humidified incubator for 72 h without shaking. For treatment, the supernatant was carefully removed and 200 µL of LB medium containing CSPs, glucose and PenStrep antibiotic mixture was added at the indicated concentrations and combinations.

Treatment for the experiments during biofilm formation was performed in the same way, except that 20 µL of a bacterial solution containing 2.5 × 10^6^ cells/mL was added instead of prepared biofilm. The lid of the 96-well plate was closed, sealed with parafilm and incubated in a humidified incubator at 37 °C without shaking for 24, 48 or 72 h, as indicated. Samples were analyzed for biofilm formation using crystal violet assay, MTT assay for metabolic activity or spot plating for cell viability, as described below.

### Crystal violet-assay

To assess the biofilm formed, the supernatant was carefully removed, and the biofilm was fixed with 200 µL of 100% ethanol for 2 min. Ethanol was removed, and samples were dried for 10 min with the lid open under the sterile work bench. Afterwards, 200 µL 0.05 (wt/vol)% crystal violet dye dissolved in sterile PBS were added and incubated at room temperature. Dye was removed after 2 min, the stained biofilm was gently washed 5 times with 200 µL sterile PBS and microscopic images were taken. For quantification, the remaining PBS was removed, and samples were dried over night with the lid open under the sterile work bench. Then, 100 µL 100% ethanol was added and incubated for 10 min at room temperature. The released dye was quantified by measuring the absorbance at 595 nm in a plate reader.

### MTT-assay

To assess metabolic activity of bacteria in the remaining or newly formed biofilm, the supernatant was carefully removed and replaced by 1 mg/mL MTT in LB medium and incubated for 30 min at 37 °C. MTT solution was removed, and samples were washed three times with 200 µL PBS, without removing the biofilm. Then, 100 µL DMSO was added and incubated at 37 °C for 30 min. The amount of metabolic active bacteria was quantified by measuring the absorbance at 570 nm in a plate reader and compared to the respective vehicle controls.

### Spot plating—CFU

To assess the cell viability of bacteria, the supernatant of treated biofilm samples was removed and the biofilm was washed with 200 µL sterile PBS. Then, 200 µL soybean casein digest lecithin polysorbate broth (SCDLP) was added, incubated at 37 °C for 15 min, and pipetted up and down at least ten times to remove biofilm^53^. A serial dilution of suspended biofilm was prepared in SCDLP, and 5 µL of each dilution were spot plated to a 60 mm petri dish filled with LB agar as described by Wang et al.^54^ and incubated overnight in a humidified incubator at 37 °C. Colony forming units (CFU) were counted and CFU/cm^2^ were calculated in relation to the area of biofilm tested. For treated non-biofilm samples, the dilution series was prepared directly from the sample in LB medium and analyzed as described.

### Statistical analysis

Measurements for biological assessment were repeated with three biological replicates (n = 3) and expressed as mean ± standard deviation (SD), unless stated otherwise. Calculations of statistical significance were performed with GraphPad Prism 8 (GraphPad Software, San Diego, USA). A two-way ANOVA and Dunnett post-hoc test were used unless otherwise indicated.

## Supplementary Information

Supplementary Figures.

## Data Availability

All data generated or analysed during this study are included in this published article (and its Supplementary Information files).

## Abbreviations

AR: Antibiotic resistance
CSPs: Carbasugar precursors
p-TsOH: P-Toluenesulfonic acid
hMSC: Human mesenchymal stem cells
HUVEC: Human umbilical vein endothelial cells
VC: Vehicle control
CV: Crystal violet
MTT: 3-(4,5-Dimethylthiazol-2-yl)-2,5-diphenyltetrazolium bromide
PenStrep: Penicillin and streptomycin
QS: Quorum sensing
agr: Accessory gene regulator
AHL: *N*-Acyl l-homoserine lactone
FAO: Fatty acid oxidation
DCFDA: 2′,7′-Dichlorofluorescin diacetate
NAC: *N*-Acetylcysteine
BHA: Butylated hydroxyanisole
