# Supplementary material for: Synergy of R-(–)carvone and cyclohexenone-based carbasugar precursors with antibiotics to enhance antibiotic potency and inhibit biofilm formation
Source: Sci Rep. 2022 Oct 26;12:18019. doi: 10.1038/s41598-022-22807-8 (PMC9606123; doi:10.1038/s41598-022-22807-8)
Supplement: Supplementary file 1 — Supplementary Figures. [file 41598_2022_22807_MOESM1_ESM.docx]

Supporting information:

Synergy of R-(-)Carvone and Cyclohexenone-based Carbasugar Precursors with Antibiotics to Enhance Antibiotic Potency and Inhibit Biofilm Formation.

Oliver Riester ^1,2^, Pia Burkhardtsmaier ^1^, Yuna Gurung ^1^, Stefan Laufer ^2,3^, Hans-Peter Deigner ^1,4,5,#^, Magnus S. Schmidt ^1,*,#^

^1^ Institute of Precision Medicine, Furtwangen University, Jakob-Kienzle-Strasse 17, Villingen-Schwenningen, 78054, Germany

^2^ Institute of Pharmaceutical Sciences, Department of Pharmacy and Biochemistry, Eberhard-Karls-University Tuebingen, Auf der Morgenstelle 8, Tuebingen, 72076, Germany

^3^ Tuebingen Center for Academic Drug Discovery & Development (TüCAD2), 72076 Tuebingen, Germany

^4^ Faculty of Science, Eberhard-Karls-University Tuebingen, Auf der Morgenstelle 8, Tuebingen, 72076, Germany

^5^ EXIM Department, Fraunhofer Institute IZI (Leipzig), Schillingallee 68, Rostock, 18057, Germany

* Corresponding author: smag@hs-furtwangen.de

# Both authors share senior authorship

Table of content

**Biocompatibility -** Figure S1 | Biocompatibility of the compounds **rac-7** and **3**. S2

**Microbial growth -** Figure S2 | Effects of the compounds rac-7 and 3 on the bacterial growth S3

**Biofilm** - Figure S3 | Evaluation of biofilm formation in the presence of compounds rac-7 and 3. S4

**NMR Spectra** - Figure S4 | ^1^H-NMR spectrum of compound **2.**  S5

**NMR Spectra** - Figure S5 | ^13^C-NMR spectrum of compound **2**. S5

**NMR Spectra** - Figure S6 | ^1^H-NMR spectrum of compound **3**. S6

**NMR Spectra** - Figure S7 | ^13^C-NMR spectrum of compound **3**. S6

**NMR Spectra** - Figure S8 | COSY spectrum of compound **3**. S7

**NMR Spectra** - Figure S9 | NOESY spectrum of compound **3** with indicated crosscoupling. S7

**NMR Spectra** - Figure S10 | ^1^H-NMR spectrum of compound **6**. S8

**NMR Spectra** - Figure S11 | ^13^C-NMR spectrum of compound **6**. S8

**NMR Spectra** - Figure S12 | ^1^H-NMR spectrum of compound **7**. S9

Biocompatibility


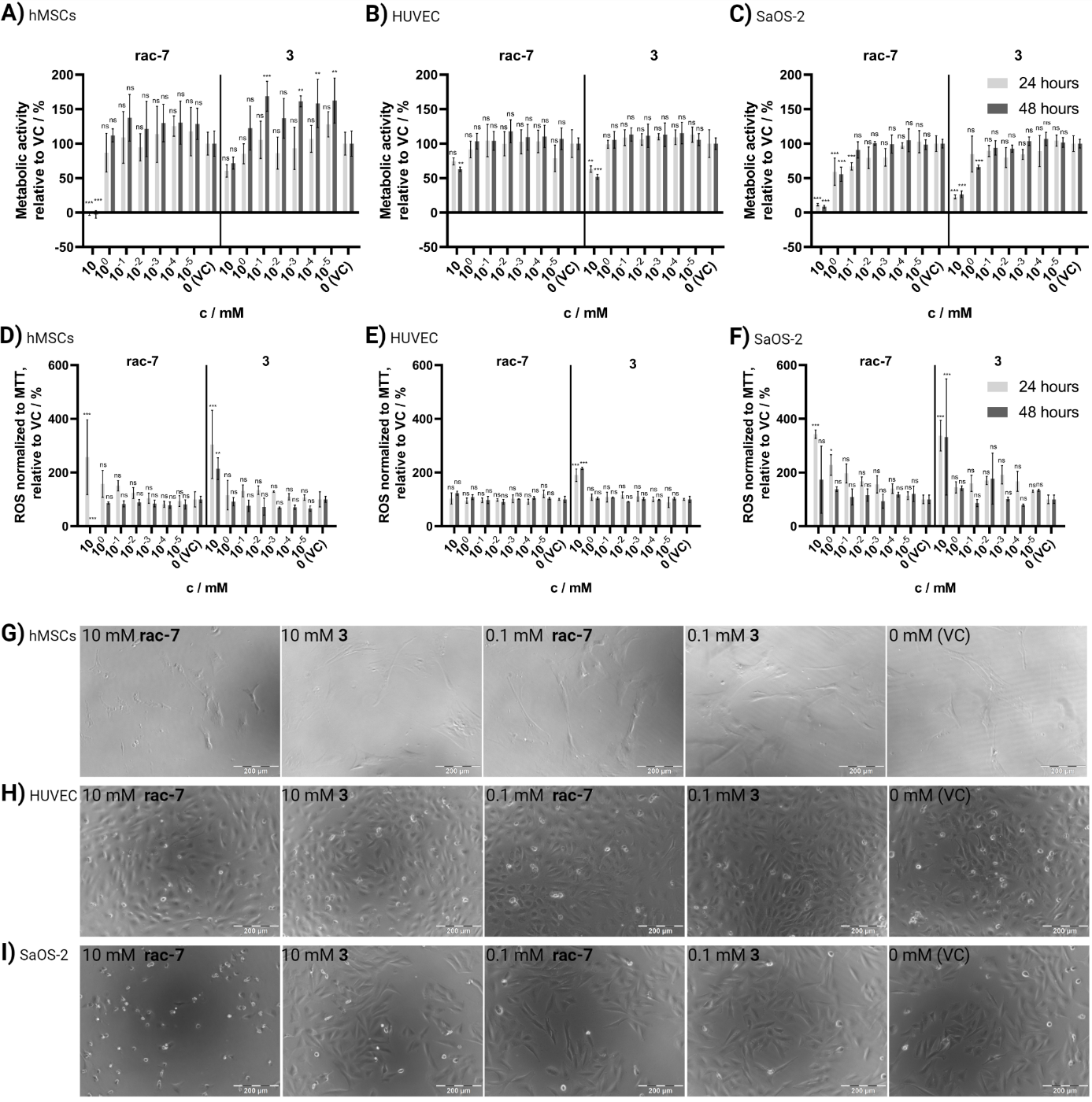


Figure S1 | Biocompatibility of the compounds **rac-7** and **3**. Human mesenchymal stem cells (hMSC), human umbilical vein endothelial cells (HUVEC) and SaOS-2 human osteogenic sarcoma cells treated for 24 and 48 hours. **A** - **C** Evaluation of metabolic activity at different concentrations of CSPs with MTT – assay in comparison to vehicle control (VC). **D** – **F** Cellular reactive oxygen species (ROS) normalized to MTT – assay and compared to VC. VC was treated with same volume of PBS without CSPs. Values are shown as mean ± SD (n = 3). Statistical significance was analyzed with Two-way ANOVA and Dunnett post-hoc test against respective VC (ns, not significant; * p < 0.05; ** p < 0.01; *** p < 0.001). **G** – **I** Representative microsopic images of hMSCs, HUVECs and SaOS-2 cells treated with compounds **rac-7** or **3**. Scale bar measures 200 µm.

Microbial growth


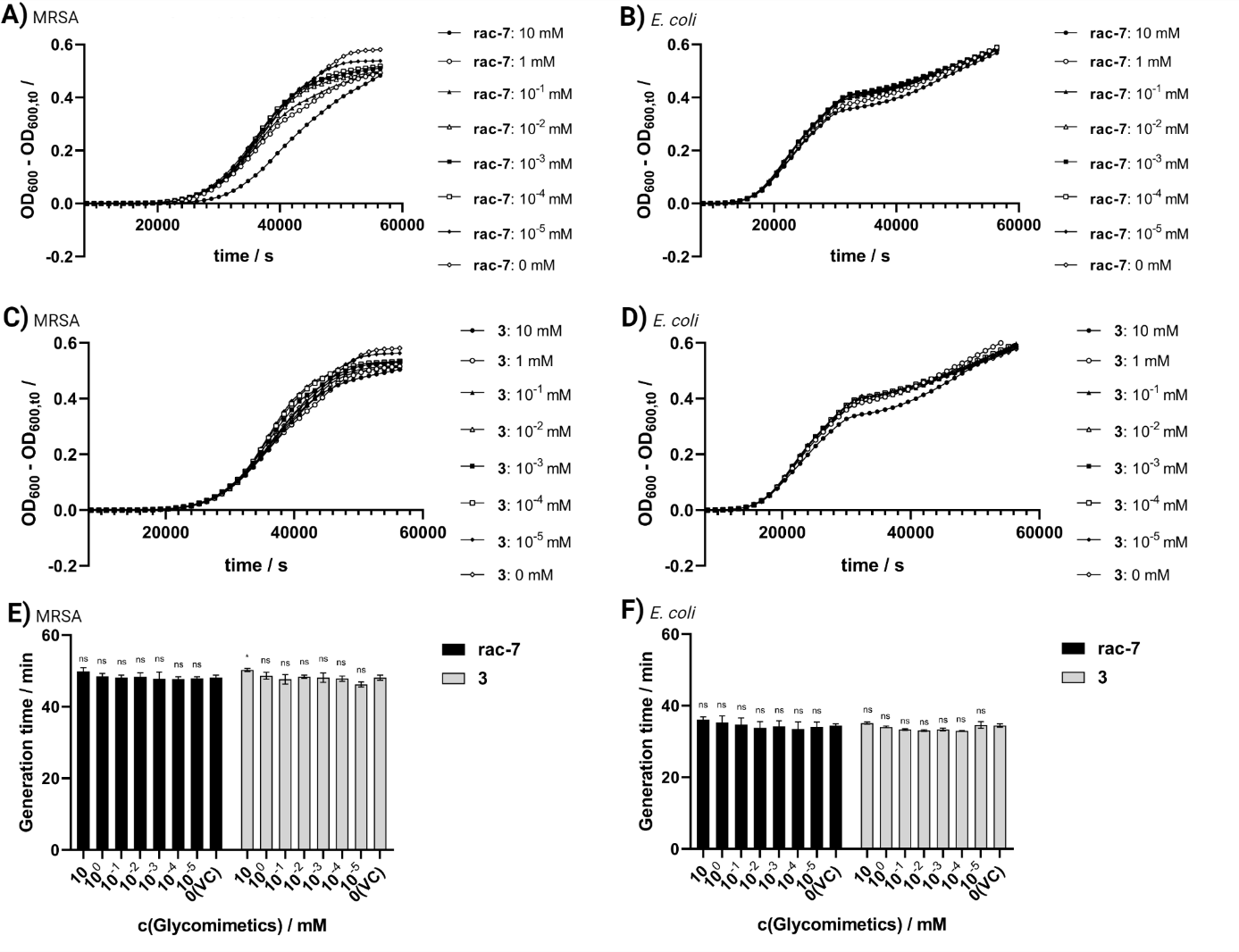
 Figure S2 | Effects of the compounds **rac-7** and **3** on the bacterial growth of *Escherichia coli* (*E. coli*; **B**, **D**, **F**) and methicillin-resistant *Staphylococcus aureus* (MRSA; **A**, **C**, **E**). Measured optical density at 600 nm (OD_600_) showing the growth curves treated with several concentrations of the carbasugar precursors (CSPs) **rac-7** (**A**, **B**) and **3** (**C**, **D**) in LB medium. Concentration-dependent influence of CSPs on the generation time of *E. coli* (**F**) and MRSA (**E**) in LB medium (additional concentrations to Figure 3). Values are shown as mean (**A - D**: n = 3) or mean ± SD (**E**, **F**: n = 3). Statistical significance was analyzed with Two-way ANOVA and Dunnett post-hoc test against respective vehicle control (VC) (ns, not significant; * p < 0.05; ** p < 0.01; *** p < 0.001).

Biofilm


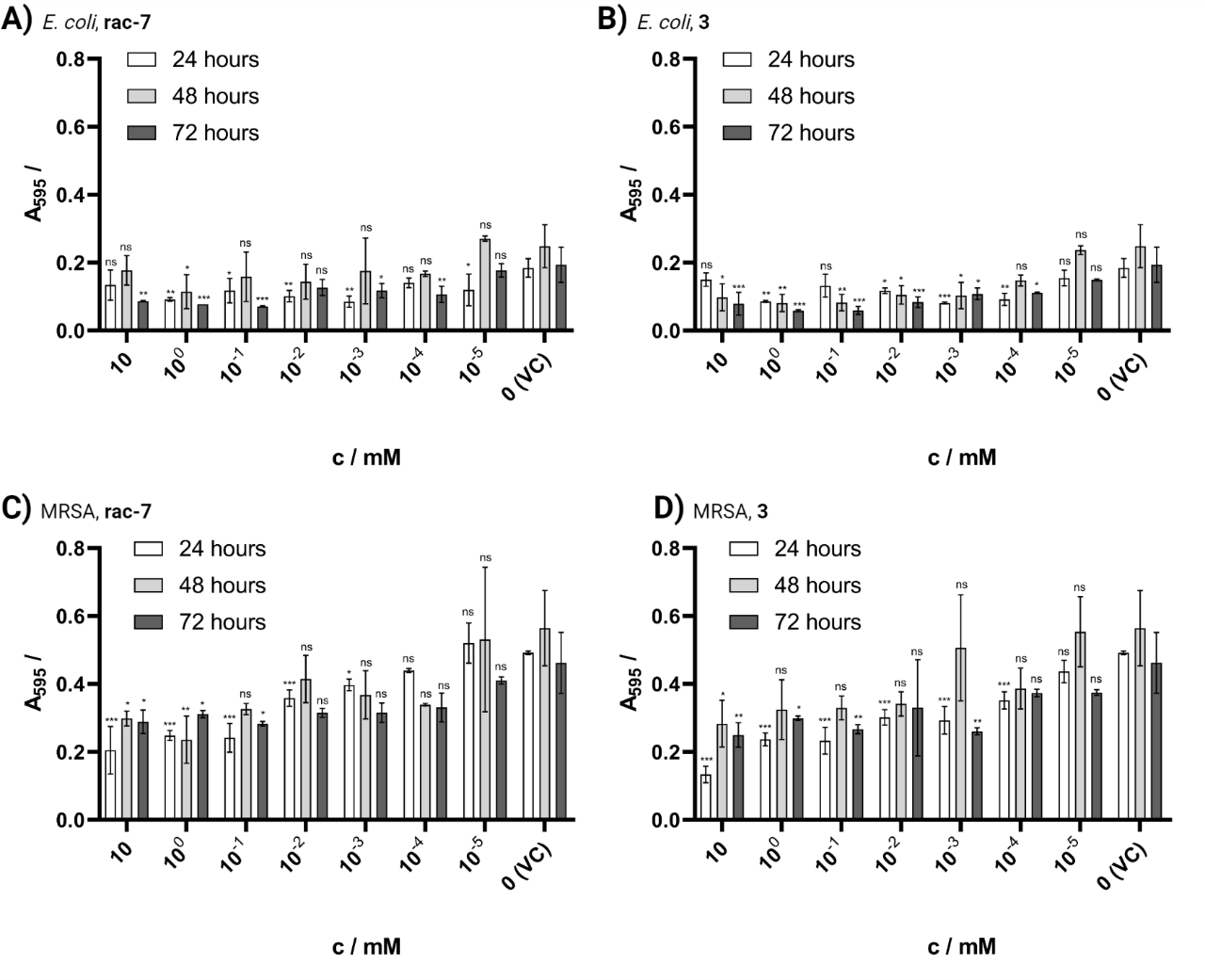


Figure S3 | Evaluation of biofilm formation in the presence of compounds **rac-7** and **3**. Biofilm formation of *Escherichia coli* (*E. coli*; **A** – **B**) and methicillin-resistant *Staphylococcus aureus* (MRSA; **C** – **D**) after 24, 48 and 72 hours of incubation in the presence of 10 mM to 10 nM carbasugar precursors. Absolute values are shown as mean ± SD (n = 2). Statistical significance was analyzed with Two-way ANOVA and Dunnett post-hoc test against respective vehicle control (n s, not significant; * p < 0.05; ** p < 0.01; *** p < 0.001).

NMR Spectra


Figure S4 | ^1^H-NMR spectrum of compound **2.**

Figure S5 | ^13^C-NMR spectrum of compound **2.**

Figure S6 | ^1^H-NMR spectrum of compound **3.**

Figure S7 | ^13^C-NMR spectrum of compound **3.**

Figure S8 |COSY spectrum of compound **3.**

Figure S9: NOESY spectrum of compound 3 with indicated crosscoupling.

Figure S10 | ^1^H-NMR spectrum of compound **6.**

Figure S11 | ^13^C-NMR spectrum of compound **6.**

Figure S12 | ^1^H-NMR spectrum of compound **7.**
